# Repurposing diacerein for the treatment of chronic wounds in recessive‐dystrophic epidermolysis bullosa patients by modulating matrix metalloproteinase‐9 expression

**DOI:** 10.1111/1346-8138.17621

**Published:** 2025-01-24

**Authors:** Sonja Dorfer, Michael Ablinger, Monika Wimmer, Julia I. Hummel, Selma Ibrahimpašić, Anja Diem, Martin Laimer, Stefanie Gruner, Josefina Piñón Hofbauer, Christina Guttmann‐Gruber, Ulrich Koller, Iris K. Gratz, Johann W. Bauer, Roland Zauner, Verena Wally

**Affiliations:** ^1^ Department of Dermatology and Allergology, EB House Austria, Research Program for Molecular Therapy of Genodermatoses University Hospital of the Paracelsus Medical University Salzburg Salzburg Austria; ^2^ Department of Biosciences and Medical Biology University of Salzburg Salzburg Austria; ^3^ Department of Dermatology and Allergology University Hospital of the Paracelsus Medical University Salzburg Salzburg Austria

**Keywords:** diacerein, matrix metalloproteinase, RDEB, wound healing

## Abstract

Recessive dystrophic epidermolysis bullosa (RDEB) is caused by mutations in *COL7A1*, leading to loss or dysfunction of type‐VII collagen (C7), a protein essential for skin stability. Clinically, patients suffer from severe skin blistering, chronic or recurrent wounds, and scarring, which predispose to early onset of aggressive squamous cell carcinoma. Previous studies showed that RDEB‐keratinocytes (RDEB‐KC) express high levels of matrix‐metalloproteinase 9 (MMP‐9), a molecule known to play a crucial role in wound chronification if dysregulated. We investigated the potential of diacerein, a small molecule that interferes with the MMP‐9 regulatory pathway, to improve wound healing in a 5‐year old RDEB patient presenting with chronic, generalized skin involvement unresponsive to previous treatment approaches. Upon 4 weeks of topical therapy applied to the patient's back, parents reported a nearly complete wound closure and a significant increase in quality of life. We also provide evidence that diacerein treatment of patient keratinocytes results in a downregulation of MMP‐9 expression, accompanied by a reduction in their ability to degrade a fibrinogen matrix. These data characterize diacerein as a potential candidate for improving wound healing in RDEB through its impact on inflammatory as well as epithelial cells.

## 1 INTRODUCTION

Patients with recessive‐dystrophic epidermolysis bullosa (RDEB) suffer from generalized, severe skin blistering and chronic or recurrent wounds. Disruption of the epithelial barrier further elicits induction of secondary inflammatory pathways, which interfere with wound healing capacities, aggravate disabling symptoms like itch and pain, and promote tissue scarring, remodeling, and carcinogenesis. Early onset of aggressive squamous cell carcinoma represents the primary cause of death in this cohort.^1^ Against this background, there is a pressing need to improve wound healing in RDEB patients, as this would prevent several secondary complications that put an extremely high clinical and psychological burden on patients.^2^

Diacerein, with its active metabolite, rhein, is a small molecule drug that is approved for the treatment of rheumatoid arthritis, where it exhibits anti‐inflammatory effects by interfering with interleukin‐1ß (IL‐1ß) signaling. Its mechanism of action (MoA) and its easy and ready availability at clinical grade, the latter being a general advantage of small molecule drugs over biologics, renders it an interesting candidate for the treatment of various inflammatory diseases, including psoriasis and epidermolysis bullosa simplex (EBS). While for psoriasis, in vitro and murine data as well as a case study (oral administration) are available,^3^, ^4^, ^5^ the development of a diacerein‐containing ointment for EBS has advanced to phase‐II/III clinical trials,^6^, ^7^ where it showed improvement of skin blistering and an excellent safety profile.

In contrast to EBS, which is mainly caused by mutations in keratins 5 or 14, RDEB is caused by mutations in the collagen‐7 (*COL7A1*) gene, leading to loss of anchoring fibrils at the dermal‐epidermal junction (DEJ). Despite major differences in severity and clinical presentation between EBS (predominantly characterized by epidermal blistering^8^) and RDEB, both subtypes show skin inflammation with IL‐1ß being a key player in the respective pathologies. Therefore, given the known MoA and the good safety profile of a 1% diacerein ointment, we here investigated its potential to improve wound healing in a case study of a patient suffering from severe RDEB.^9^, ^10^, ^11^ In addition, to elaborate on potential mechanisms underlying impaired RDEB‐wound healing reported in existing literature, we further explored the role of keratinocytes and their response to diacerein. This is in view of their well‐known immune functions, including the expression of and interplay with inflammatory mediators, growth factors, and matrix metalloproteinases (MMPs). In general, increased levels of MMPs and pro‐inflammatory cytokines (e.g., IL‐1ß), are common to chronic wounds and are even regarded as wound status biomarkers.^12^ MMP‐9, in particular, has been found to be indicative of inflammation and to inversely correlate with wound healing rates, with keratinocytes being a major contributor of this protease.^13^, ^14^

Here, we present a case report of a patient with RDEB who received 1% diacerein ointment to treat multiple lesions covering a large area of her back. Given the availability of keratinocytes from this patient, we further aimed to shed light on diacerein's MoA in RDEB, specifically considering the role of MMP‐9 in wound healing.^9^, ^10^, ^11^, ^15^, ^16^, ^17^

## 2 METHODS

### 2.1 Patient

A five‐year old patient genetically diagnosed with severe RDEB (c.425A > G/p.K142R; leading to absence of C7 protein due to aberrant splicing,^18^) presented with extensive wounding and erosions on her upper back. Skin lesions mainly included chronic open wounds (stable and refractory, not healing >4 weeks) as well as recurrent wounds due to repetitive blistering (healing with subsequent reopening). The patient was treated with 1% diacerein ointment for 4 weeks on her back in a named patient program (NPP). The ointment was applied with every dressing change, during which photographs were taken by the parents. Photographs and information on potential adverse events as well as any potential effects of the treatment were provided by the parents by e‐mail.

Permission for publication of the case details and images was obtained from the patient's parents.

### 2.2 Cell lines

Primary and human papilloma virus 16 E6/E7 immortalized RDEB and healthy control keratinocytes (HC‐KC) were cultured in defined, serum‐free CnT‐Prime Epithelial Culture Medium (CnT‐PR, CELLnTEC) at 37°C with 5% CO_2_ in a humidified incubator. Cells were tested to be negative for mycoplasma infection before experiments. Prior to cell stimulation, the medium was changed to CnT‐Prime Epithelial Homeostasis Medium (CnT‐PR‐H, CELLnTEC), which is low in growth factors, to increase the responsiveness to experimental stimuli. Cell line characteristics are shown in Table S1.

### 2.3 Immunofluorescence

Formalin‐fixed paraffin embedded sections were deparaffinized and stained as described elsewhere.^19^ Regions (wound margins, non‐lesional skin, denuded skin) were defined upon consultation with the clinic's histopathologist. All antibodies used are listed in Table S2. Imaging was performed on a Zeiss LSM710 confocal microscope, and ImageJ^20^ was employed to quantify the mean fluorescence intensity of different regions of interest (ROI), normalized by area.

### 2.4 Diacerein treatment

First, 5 × 10^5^ cells were seeded in 6‐well plates and cultured overnight, before the cells were treated with 10 μg/mL diacerein (D9302, Sigma Aldrich) dissolved in medium. Cells and supernatants for protein extraction were harvested 24 h post treatment.

### 2.5 Whole cell lysate

Cells grown to 80% confluence were lysed in radio‐immunoprecipitation buffer (RIPA, sc‐24 948, Santa Cruz Biotechnology) supplemented with phosphatase inhibitors (5870S, Cell Signaling). Cells were homogenized by pipetting three times through a 27G syringe needle and then centrifuged for 10 min at 4°C and 10 000*g* to remove cell debris. Protein concentrations were measured using a bicinchoninic acid Assay (23227, Thermo Fisher).

### 2.6 Protein from cell culture supernatant

Proteins from conditioned medium were precipitated by acetone. Briefly, supernatant was centrifuged for 10 min at 1000*g* to remove dead cells and cell debris. One volume of supernatant was then incubated with 4 volumes of ice‐cold acetone overnight at −20°C. Subsequently, samples were centrifuged for 30 min at 8000*g* and the supernatant removed. Residual acetone was allowed to evaporate for 30 min at room temperature. The protein pellet was resuspended in RIPA buffer and the protein content determined as described above.

### 2.7 Western blot

Proteins were incubated with a 4x Laemmli sample buffer (161074, Biorad) for 5 min at 95°C and separated on Mini‐PROTEAN® TGX Stain‐Free™ Protein Gels (4568123, BioRad). Total protein loaded per well was visualized on gels by a stain‐free UV‐induced reaction (ChemiDoc™ MP, Biorad). Proteins were transferred onto 0.45 μm polyvinylidene difluoride membranes (1704274, Biorad) using the Trans‐Blot® Turbo™ Transfer System (1704150, Biorad). After blocking the membranes with 5% bovine serum albumin (BSA), dissolved in tris‐buffered saline (TBS) for 1 hour at room temperature, the membranes were incubated overnight at 4°C with primary antibodies diluted in 5% BSA/TBS supplemented with 0.02% sodium dodecyl sulphate. Membranes were washed with TBS with 0.1% Tween‐20 aqueous solution (Thistle Scientific) and incubated with secondary antibodies for 1 hour at room temperature. After washing, the membranes were imaged and analyzed using the ChemiDoc™ MP and Image Lab™ Software (version 6.0.1, Biorad). Protein expression in supernatants was normalized to correspond to total cellular protein. The antibodies used are listed in Table S3.

### 2.8 Cell stimulation

#### 2.8.1 IL‐1β stimulation

First, 2.5 × 10^5^ cells/well were seeded overnight in a fully supplemented medium into 6‐well plates; 24 h prior to stimulation, the medium was changed to basal medium. Cells were then exposed to 15 ng/mL human recombinant IL‐1β (201‐LB, R&D Systems) reconstituted in PBS containing 0.1% BSA in basal medium for 4 h or 24 h, respectively.

#### 2.8.2 Lipopolysaccharides from Pseudomonas aeruginosa stimulation

Overnight, 2 × 10^5^ cells/well were seeded in a fully supplemented medium into 6‐well plates; 24 h prior stimulation, the medium was changed to basal medium. Cells were stimulated in basal medium with 0.1, 1, 2, and 6 μg/mL LPS (L9143, Sigma‐Aldrich) reconstituted in H_2_O for 24 h.

### 2.9 Semi‐quantitative real‐time PCR

Total RNA was purified from cells using the RNeasy Mini Kit (74104, Qiagen) according to the manufacturer's instructions. Genomic DNA was digested with the RNAse‐free DNAse Set (1708890, Qiagen). For reverse transcription, the iScript™ cDNA Synthesis Kit (1708890, BioRad) was used. Semi‐quantitative real‐time polymerase chain reaction (sqRT‐PCR) was performed as described elsewhere.^19^ Primer sequences are listed in Table S4.

### 2.10 Matrix degradation assay

Recessive‐dystrophic epidermolysis bullosa 223‐KC E6/E7 cells were pre‐treated for 24 h with either 10 μg/mL diacerein (D9302, Sigma Aldrich) dissolved in medium, 6 μg/mL LPS (L9143, Sigma‐Aldrich), reconstituted in H_2_O, or a combination thereof. 10^5^ cells were subsequently seeded in CnT homeostasis medium (CnT‐PR‐H, CELLnTEC) in a matrix (matrix 1) composed of 8.4 mg/mL fibrinogen dissolved in 0.9% NaCl (F4883, Sigma Aldrich) and 0.06 National Institute of Health (NIH) units thrombin reconstituted in 25 mM CaCl_2_ (T8885, Sigma Aldrich) in the middle of a 24‐well plate (3527, Costar). After polymerization of the cell matrix, a second matrix (matrix 2) composed of 7.9 mg/mL fibrinogen, 0.2 NIH units of thrombin, 17% fetal calf serum (FCS) and CnT‐Prime Epithelial Culture Medium (CnT‐PR, CELLnTEC) was added around the outside of the cell matrix to cover the whole well. After polymerization of the second matrix, CnT Prime Medium (CnT‐PR‐H, CELLnTEC), including respective treatments (2 μg/mL diacerein, 6 μg/mL LPS and 2 μg/mL diacerein with 6 μg/mL LPS), was added on top of the matrix. Brightfield images of the whole well were acquired at 4x with the TECAN Cyto plate reader and image analysis was performed with ImageJ.

### 2.11 Statistical analysis

Analyses were performed with GraphPad Prism version 9.0.0 (GraphPad Software, San Diego, CA, USA). Data are shown as mean and standard error of mean (SEM). A *p*‐value of ≤0.05 was considered statistically significant. Details regarding the statistical analysis and number of independent replicates are described in the Figure legends.

## 3 RESULTS

### 3.1 Diacerein treatment of a patient with severe RDEB

**FIGURE 1:**
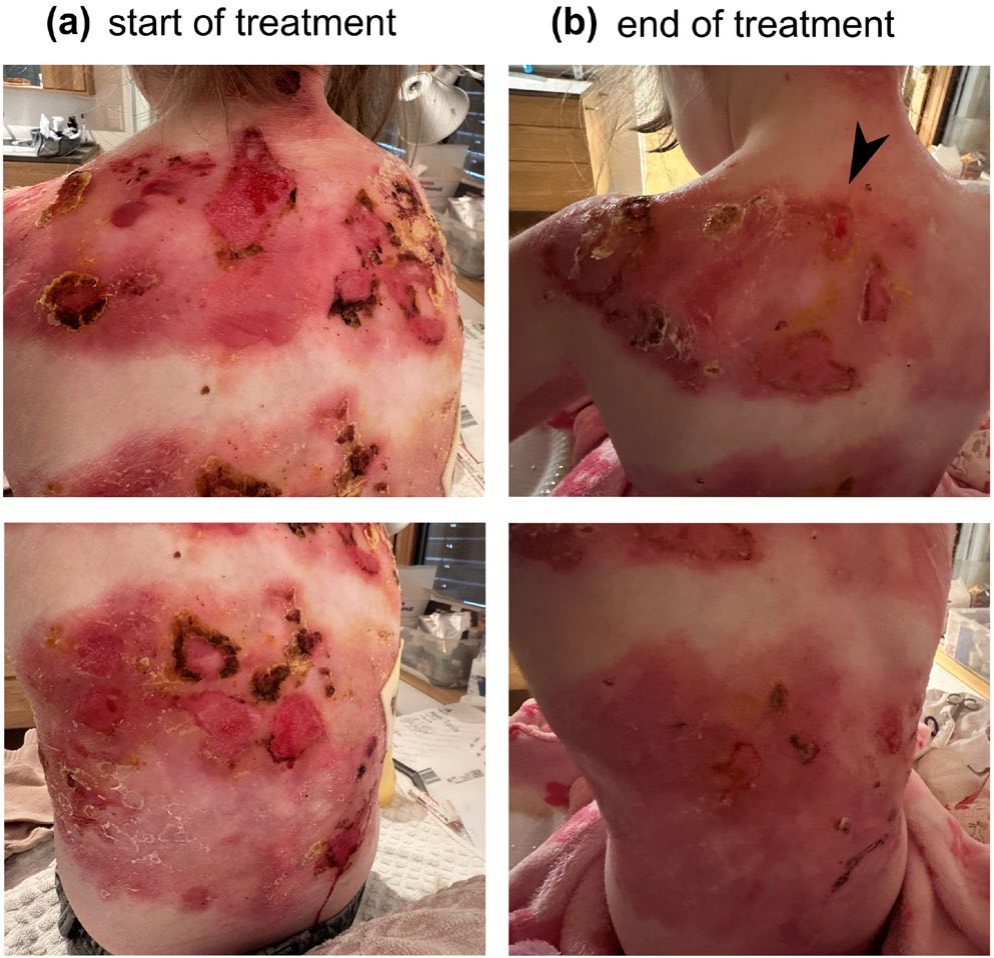
Treatment of a recessive dystrophic epidermolysis patient with diacerein. (a) Day 1 when starting the treatment with 1% diacerein, the patient presented with extensive wounding on the whole back. (b) After 28 days of treatment, all lesions but one (arrow) closed.

A 5‐year‐old patient suffering from RDEB presented with extensive wounding and erosions on her upper back. According to the parents, some wounds had been present for more than 4 weeks, while in other areas wounds healed and recurred. In the course of an NPP, the patient was given 1% diacerein cream to apply once daily on her upper back during regular wound dressing change. After only 2 days, an overall amelioration of the treated skin was reported. Given the fast success of the treatment, the family decided to extend the treatment area also to the lower back (Figure 1a). After 4 weeks, the patient's mother reported that all wounds, with the exception of one small, coin‐sized lesion, had closed (Figure 1b). In addition, the patient experienced a considerable increase in quality of life, which was attributed to reduced itch, increased quality of sleep accompanying a cessation of bedwetting, an increase in energy during the day, no adhesion of the pajama to the skin in the morning, and a notable reduction in the use of dressings. In addition, erythema decreased. Of note, these observations were reported by the mother and were not quantified. After an additional 6 weeks the mother informed us that the child's back had sustainably improved, with only a single ~2.5 cm^2^ lesion. “*Otherwise, the back is beautiful, and the skin is recovering very well*” (comment by the patient's mother).

These observations prompted us to investigate the mechanisms by which diacerein might promote wound re‐epithelialization, focusing specifically on the modulation of the IL‐1ß—MMP‐9 axis in RDEB keratinocytes, especially given that (i) diacerein is known to act on the IL‐1ß signaling pathway, (ii) MMP‐9 is a downstream target of the IL‐1ß signaling pathway and IL‐1ß was shown to be upregulated in fibroblasts isolated from chronic RDEB‐wounds,^21^ (iii) MMP‐9 overexpression has also been demonstrated in cultured RDEB‐KC;^22^ (iv) a constitutive overexpression of MMPs has been described to contribute to aberrant wound healing and wound chronification in general,^23^ and (v) MMP‐9 was shown to be highly abundant in blister fluids of RDEB patients.^24^

Based on the above, we hypothesized that RDEB keratinocytes would respond to diacerein treatment by downregulation of MMP‐9, which is a key event in facilitating wound closure.

### 3.2 Induction of IL‐1ß and MMP‐9 in RDEB‐KC upon rIL‐1ß and LPS stimulation

**FIGURE 2:**
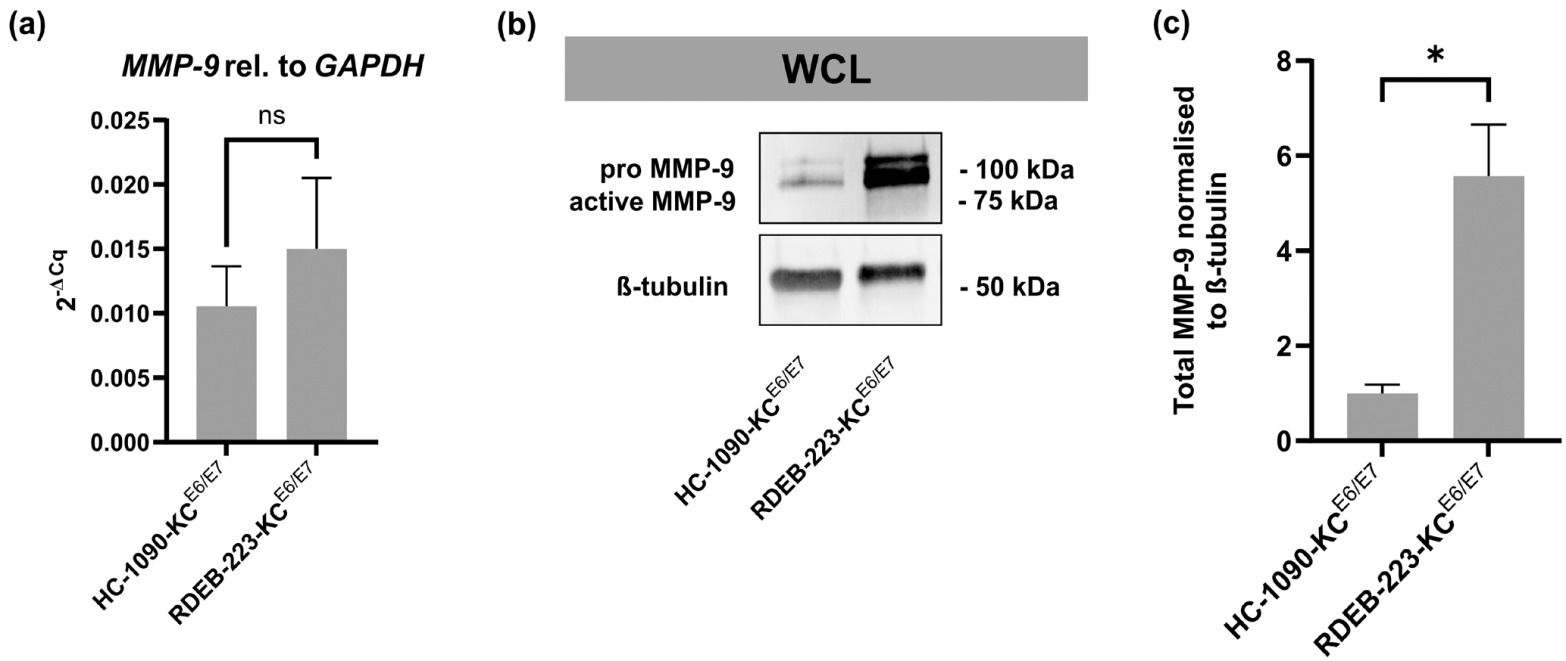
Baseline expression levels of matrix‐metalloproteinase 9 (MMP‐9) in healthy control keratinocytes (HC‐1090‐KC^E6^ ^/^ ^E7^) and recessive dystrophic epidermolysis bullosa keratinocytes (RDEB‐223‐KC^E6^ ^/^ ^E7^) on RNA and protein level. (a) Expression levels of MMP‐9 given relative to glyceraldehyde 3‐phosphate dehydrogenase are shown as 2^−ΔCq^. (b) Representative Western blot showing MMP‐9 in whole cell lysates (WCL) of HC‐1090‐KC^E6^ ^/E7^ and RDEB‐223‐KC^E6^ ^/E7^ cells. The MMP‐9 upper band indicates the pro‐ (~92 kDa) and the lower band indicates the active form (~83 kDa) of MMP‐9. (c) Dosimetric quantification of MMP‐9 abundance in WCLs. Western blot data were normalized to ß‐tubulin, calculated as fold‐change RDEB‐ versus HC‐KC. Bar plots represent mean and standard error of mean (SEM) from 3 to 5 independent replicates. Differences between groups were analyzed using an unpaired, two‐sided *t*‐test. Non‐significant (ns): *p* > 0.05, *p ≤ 0.05.

We first analyzed the level of MMP‐9 expression in keratinocytes isolated from a skin biopsy previously taken from the NPP‐patient when she was 1‐year old (RDEB‐223‐KC). We observed a non‐significant increase in expression levels of MMP‐9 by (sq)RT‐PCR in RDEB‐223‐KC as compared to keratinocytes derived from a 23‐year old healthy donor (HC‐1090‐KC, Figure 2a). Notably, at the protein level, both the pro‐ and active‐forms of MMP‐9 were significantly increased in RDEB‐223‐KC, indicating a difference in regulation of MMP‐9 levels at the post‐transcriptional level (Figure 2b,c). In addition, analyses of IL‐1ß and other members of the IL‐1ß signaling cascade, namely IL‐1‐receptor (IL‐1R) and IL‐1‐receptor antagonist (IL‐1RA), in a set of RDEB‐KC lines, showed no differential expression on the mRNA level. This was also true for patient‐derived RDEB‐223‐KCs, except for IL‐1RA, which was significantly downregulated in this cell line (Figure S1). Also, in this additional set of RDEB‐keratinocytes, MMP‐9 expression was not significantly increased by (sq)RT‐PCR (Figure S1).

We next tested whether MMP‐9 expression could be enhanced by exogenous stimuli that are known to contribute to wound chronification. As RDEB‐wounds are usually severely infected and bacterial toxins stimulate inflammation and play a role in sustaining low‐grade unresolved inflammation, we used LPS to trigger an inflammatory response in the patient's keratinocytes.^25^, ^26^, ^27^ Treatment of RDEB‐223‐KC with LPS induced a dose‐dependent increase in both IL‐1β and MMP‐9 transcription in vitro. At 6 μg/mL LPS, mRNA levels of IL‐1β (fold change [FC] = 2, *p* = 0.023) and MMP‐9 had increased significantly (FC = 5, *p* = 0.004) (Figure S2). Of note also, exogenous stimulation with 15 ng/mL recombinant (r)IL‐1ß resulted in an increase in mRNA levels of IL‐1ß (4 h: FC = 9, *p* = 0.039, 24 h: FC = 2, *p* > 0.1) and MMP‐9 (4 h: FC = 7, *p* = 0.04, 24 h: FC = 7, *p*‐value 0.014), respectively (Figure S3).

### 3.3 The anti‐inflammatory drug diacerein modulates MMP‐9 expression in RDEB‐KC

**FIGURE 3:**
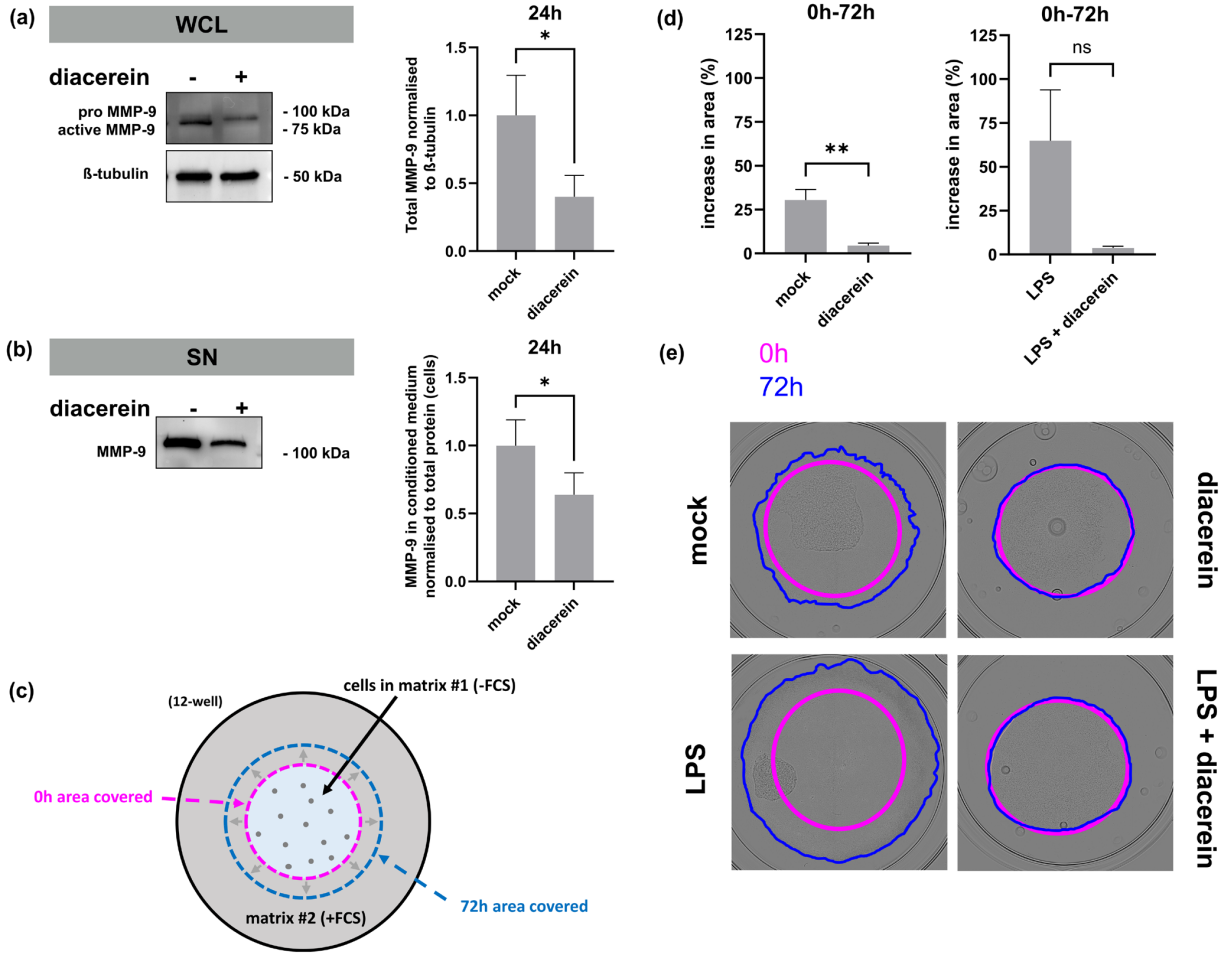
Modulation of matrix‐metalloproteinase 9 (MMP‐9) expression upon diacerein treatment in recessive dystrophic epidermolysis keratinocytes (RDEB)‐223‐KC^E6^ ^/E7^. Representative Western blots show the levels of MMP‐9 expression in (a) whole cell lysates and (b) conditioned medium (supernatant: SN) of RDEB‐223‐KC^E6^ ^/E7^ cells, either mock treated or treated for 24 h with 10 μg/mL diacerein. The upper band indicates the pro‐ (~92 kDa) and the lower band indicates the active form (~83 kDa) of MMP‐9. Data were normalized to ß‐tubulin and relative to levels in mock treated cells. Protein expression in supernatants was normalized to the total protein of harvested cells, approximating the number of cells where the conditioned medium derived from. Statistical analysis was performed using a paired *t*‐test, considering *p* < 0.05 as statistically significant, with non‐significant (ns): *p* > 0.05, *p ≤ 0.05. Bars represent mean and error bars standard error of mean (SEM) of (a) *n* = 5 and (b) *n* = 9 independently performed biological replicates. (c) Schematic showing matrix degradation assay. (d, e) RDEB‐223‐KC^E6^ ^/E7^ were either pre‐treated for 24 h with vehicle control (mock) or 10 μg/mL diacerein, 6 μg/mL Lipopolysaccharides (LPS), or a combination thereof. Brightfield images of the whole well were acquired at 0 and 72 h post‐treatment and image analysis was performed with ImageJ. The degree of matrix degradation was measured as the percentage increase in area covered by cells 72 h post‐treatment (blue border) compared to area at 0 h (pink border). Bars show mean, error bars show standard error of mean (SEM) of increase in area (%) from 3 to 5 independent experiments. An unpaired *t*‐test was used for statistical analysis. Non‐significant (ns): *p* > 0.05, **p ≤ 0.01. Representative images are shown.

To investigate whether diacerein could modulate MMP‐9 expression in patient keratinocytes, RDEB‐223‐KC were incubated with 10 μg/mL diacerein for 24 h. Compared to mock‐treated cells, MMP‐9 protein levels decreased significantly in diacerein‐treated RDEB‐223‐KC (*p*‐value 0.013) (Figure 3a). Similarly, in conditioned medium, MMP‐9 was also significantly reduced upon diacerein treatment (*p*‐value 0.0278) (Figure 3b). Expression levels of C7 were not affected by diacerein treatment (Figure S4). These findings suggest that the beneficial therapeutic effect of diacerein may, at least partly, be conferred by MMP‐9 modulation.

MMP‐9 is a key regulator of keratinocyte migration during wound healing, where it plays an important role in matrix degradation. Predominantly during the inflammatory phase, MMP‐9 is involved in breaking down the damaged matrix, thereby activating various growth factors as well as signaling proteins that induce cell migration, immune responses, inflammatory reactions, and, ultimately, tissue repair.^23^, ^28^ During normal wound healing, MMP‐9 levels peak 24 h post‐wounding and decrease again significantly after 48 h. However, in chronic wounds, persistently increased levels of MMP‐9 were shown to have a detrimental effect, correlating with impaired wound healing due to excessive matrix degradation.^29^, ^30^, ^31^ Thus, inhibition of MMP‐9 in the context of chronic wounding should support wound healing.

In this assay, patient keratinocytes were seeded in the center of a well and surrounded by a gel matrix. An increase in surface area covered by RDEB‐223‐KC after 72 h in culture was interpreted as the degree of MMP‐mediated matrix degradation that enables keratinocytes to spread and migrate towards a chemoattractant (Figure 3c). Pre‐treatment of RDEB‐223‐KC with diacerein resulted in a reduction of MMP‐9 levels, a decrease in matrix degradation, and fewer cells spreading into the matrix‐covered dish area as a consequence (Figure 3d,e). Notably, at 72 h, LPS‐treated patient keratinocytes covered a much larger surface area compared to mock‐treated cells (Figure 3d,e), indicative of enhanced MMP‐9 activity. Concomitant treatment with the anti‐inflammatory drug diacerein counteracted the pronounced matrix degradation seen with LPS stimulation.

### 3.4 Elevated expression of MMP‐9 at the wound margin and chronic wound of a patient with RDEB

To put our in vitro findings within an in‐situ context, we sought to confirm MMP‐9 expression in the wound edges of a chronic wound in RDEB. We analyzed both the abundance of MMP‐9 as well as IL‐1ß, as its upstream regulator and a common marker for chronic inflammation, in available tissue sections from a 31‐year‐old RDEB patient. Of note, as biopsying chronic wounds is considered particularly burdensome for patients, we were not able to obtain tissue samples of a chronic wound from the 5‐year‐old NPP patient.

**FIGURE 4:**
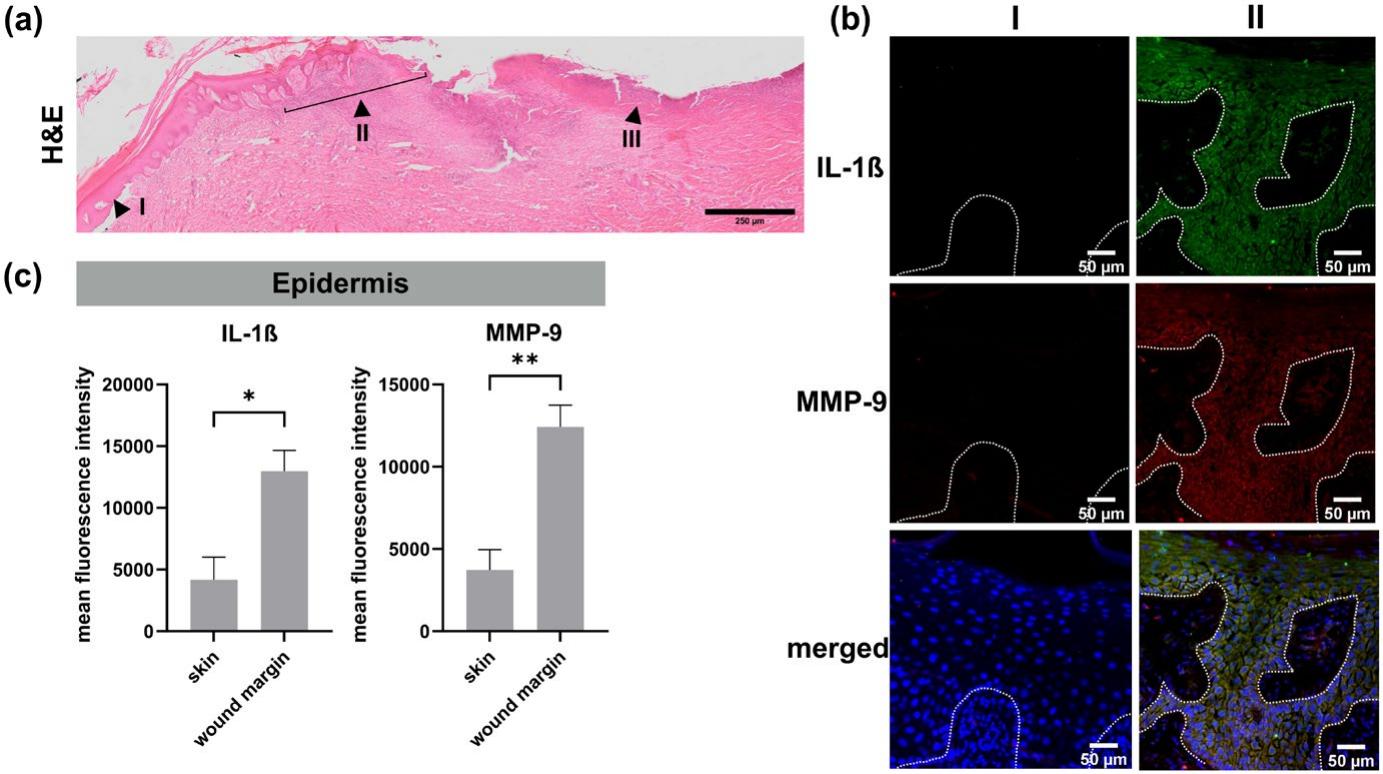
Expression profile of interleukin (IL‐)1β and matrix‐metalloproteinase 9 (MMP‐9) in recessive dystrophic epidermolysis (RDEB) skin in relation to the wound edge. (a) Hematoxylin and eosin staining of an RDEB patient skin biopsy shows the transition zone from (I) non‐lesional skin to (II) wound margin, and (III) chronic wound/denuded skin. Scale bar: 250 μM. (b) Immunofluorescence staining of the tissue sections for IL‐1β (green), and MMP‐9 (red) in combination with nuclear counterstain 4′,6‐diamidino‐2‐phenylindole, (DAPI) (blue). The dermo‐epidermal junction is indicated by white dashed lines. Scale bar: 50 μM. (c) Quantification of the mean fluorescence intensity of IL‐1β and MMP‐9 for keratinocytes of (non‐lesional) skin and keratinocytes at the wound margin, normalized by area. Differences between groups were analyzed using an unpaired, two‐sided *t*‐test. A *p*‐value of <0.05 was considered statistically significant with non‐significance (ns) at: *p* > 0.05, *p ≤ 0.05, **p ≤ 0.01.

Compared to non‐lesional regions of the same RDEB skin section, IF staining revealed a significant increase in the expression levels and co‐localization of both MMP‐9 and IL‐1β in keratinocytes of the wound margin (MMP‐9: FC = 3, *p* = 0.0056, IL‐1β: FC = 3, *p* = 0.0175). Within the dermis of denuded skin, fluorescence intensities of IL‐1ß and MMP‐9 were also increased (Figure S5). (Figure 4a–c). These observations are in line with previous data showing that, in response to inflammatory triggers such as paracrine IL‐1ß signaling from immune cells, keratinocytes express particularly high levels of MMP‐9 in a chronic wound setting, which is consequently associated with poor healing outcomes.^12^

Given the high expression of MMP‐9 at wound edges in RDEB chronic wounds (Figure 4), which is thought to contribute to a failure to progress through the various stages of wound healing in a timely manner, the ability of diacerein to reduce MMP‐9 levels, as described above, supports its potential as a therapeutic agent to treat RDEB‐associated wound healing defects.

## 4 DISCUSSION

Patients with RDEB suffer from extensive wounding due to particularly fragile skin, which is caused by mutations in the *COL7A1* gene, resulting in absence or loss‐of‐function of C7 and, consequently, impaired dermo‐epidermal anchorage. Additionally, impaired wound healing in these patients, marked by persistent inflammation with a plethora of cytokines being deregulated (e.g., transforming growth factor‐ß (TGF‐ß), IL‐6^22^, ^32^, ^33^), accumulation of mutations,^27^ and bacterial infections along with loss of microbial diversity,^34^ contribute to their poor quality of life. All these factors further predispose to the development of life‐threatening squamous cell carcinomas (SCC) in chronic wounds.^35^

Despite the progress in understanding the mechanisms related to wound chronification in RDEB, treatment options are still scarce.^36^, ^37^, ^38^ Given the rarity of EB, and RDEB in particular, patients would benefit from repurposing known drugs, as this approach comes with shorter development times because of the known safety profiles and the availability of early phase clinical trial data. This, in turn, requires less financial investment, increasing the potential to raise money for clinical developments for this rare patient population.^39^

Diacerein is a small molecule that was first approved for the treatment of osteoarthritis.^40^ Given its MoA in regulating IL‐1ß signaling, it has been investigated, repurposed, and reformulated for the treatment of severe EBS, a subtype of EB that is caused by dominant mutations in either keratin 5 or 14. These mutations lead to conformational changes and aggregation and accumulation of the affected keratins, in turn triggering a pro‐inflammatory response marked by the overexpression of the pro‐inflammatory cytokine IL‐1ß and its downstream targets (including MMP‐9), which could be attenuated by diacerein in vitro and in clinical trials.^6^, ^41^, ^42^, ^43^ Reformulation to a topically applied ointment resulted in an excellent safety profile, in contrast to the oral formulation, which was withdrawn from the market for elderly people (< 65 years), given its severe gastro‐intestinal side effects.^44^

Because of its ability to modulate expression of MMP‐9, which is dysregulated in RDEB and a known contributor to wound chronification in general, diacerein stands out as a promising drug candidate to improve wound healing in this patient group. Given the favorable safety profile of a topical formulation of diacerein, we used the 1% diacerein ointment in an NPP approach.^6^, ^7^, ^43^

Topical administration of diacerein to a 5‐year‐old RDEB patient for 4 weeks improved wound healing in the treated area and led to a nearly complete closure of chronic and recurrent wounds that had been present for several weeks before starting the treatment. The patient's parents reported a considerable improvement in the quality of life of the patient that was reflected in various aspects affecting everyday life, such as improved quality of sleep, general well‐being, etc.

Given these promising results, we sought to characterize the MoA of diacerein in RDEB. We focused here on the level of keratinocytes as potential contributors to wound chronification, given their role as producers of *COL7A1* and in immune functions such as bacterial defense. In addition, overexpression of MMPs has been observed frequently in the context of SCCs, where a high proportion of MMP‐9 was shown to be expressed by tumor keratinocytes as well as tumor‐infiltrating immune cells. In this context, MMP‐9 is a key player in activating TGF‐ß, which is constitutively expressed at increased levels in normal RDEB‐skin.^22^, ^45^

This study has several limitations including the treatment of only one patient and the short application time of the drug. Furthermore, clinical results were reported only by the patient's parents, supported by private photographs. Therefore, it will be important to test diacerein systematically in a future clinical trial in order to confirm its beneficial effect on wound healing in RDEB.

In summary, this single‐case observation indicates that diacerein was effective in promoting healing of both recurrent and long‐standing wounds of an RDEB patient within 4 weeks of treatment. We believe that these positive effects on wound healing are, at least in part, mediated by diacerein's ability to modulate the expression of MMP‐9 derived from RDEB‐keratinocytes. Certainly, given the complex nature of wound healing in general, several other cell types contribute to excessive inflammation and impaired wound healing in RDEB (e.g., fibroblasts, immune cells), and an additional effect of diacerein on those cells is likely, but was not addressed here.^32^ This is supported by our observations of reduced MMP‐9 protein levels with diacerein treatment, also in the presence of a bacterial trigger. Functionally, this translated into reduced degradation of a fibronectin matrix by RDEB‐keratinocytes treated with diacerein. These early data point to diacerein as a potential candidate for the treatment of wounds in RDEB.

## FUNDING INFORMATION

This project received financial support from DEBRA Alto Adige and DEBRA Austria.

## CONFLICT OF INTEREST STATEMENT

Verna Wally and Johann W Bauer are consultants with, and hold shares in Diaderm GmbH, a company with an interest in drug development for EB. All other authors have no conflicts of interest to declare.

## ETHICS STATEMENT

Ethical approval to collect and bank biological material was granted by the ethics committee of the county of Salzburg, Austria (415‐EP/73/192–2013).

## INFORMED CONSENT STATEMENT

Permission for publication of the case details and images was obtained from the patient's parents.

## Supporting information


**Data S1:**Supporting Information.
