# Supplementary material for: Repurposing diacerein for the treatment of chronic wounds in recessive‐dystrophic epidermolysis bullosa patients by modulating matrix metalloproteinase‐9 expression
Source: J Dermatol. 2025 Jan 24;52(3):423–31. doi: 10.1111/1346-8138.17621 (PMC11883725; doi:10.1111/1346-8138.17621)
Supplement: Supplementary file 1 — Data S1:Supporting Information. [file JDE-52-423-s001.docx]

# SUPPLEMENTAL FIGURE LEGENDS

## Fig. S1. Basal expression levels of IL-1ß and further members of the Il-1ß regulatory cascade, evaluated by semi-quantitative real-time PCR. Relative expression levels of (a, g) IL-1β, (b, h) IL-1RA, (c, i) IL-1R, (d, j) MMP-9, (e, k) MMP-13 and (f, l) TNF-alpha in normal (HC-KC) and RDEB-patient keratinocytes relative to GAPDH are shown as 2^-ΔCq^. Keratinocytes derived from the NPP-patient are shown separately (g-l) from other patient-derived keratinocytes (a-f). Results show no differential expression on the mRNA when comparing HC-KC and RDEB-KC, except for IL-1RA, that is significantly downregulated in the patient-derived keratinocytes. Bar plots represent mean and standard error of mean (SEM) from 3-5 independent, biological replicates. Differences between groups were analysed using an unpaired t-test. Ns: p > 0.05, *: p ≤ 0.05.


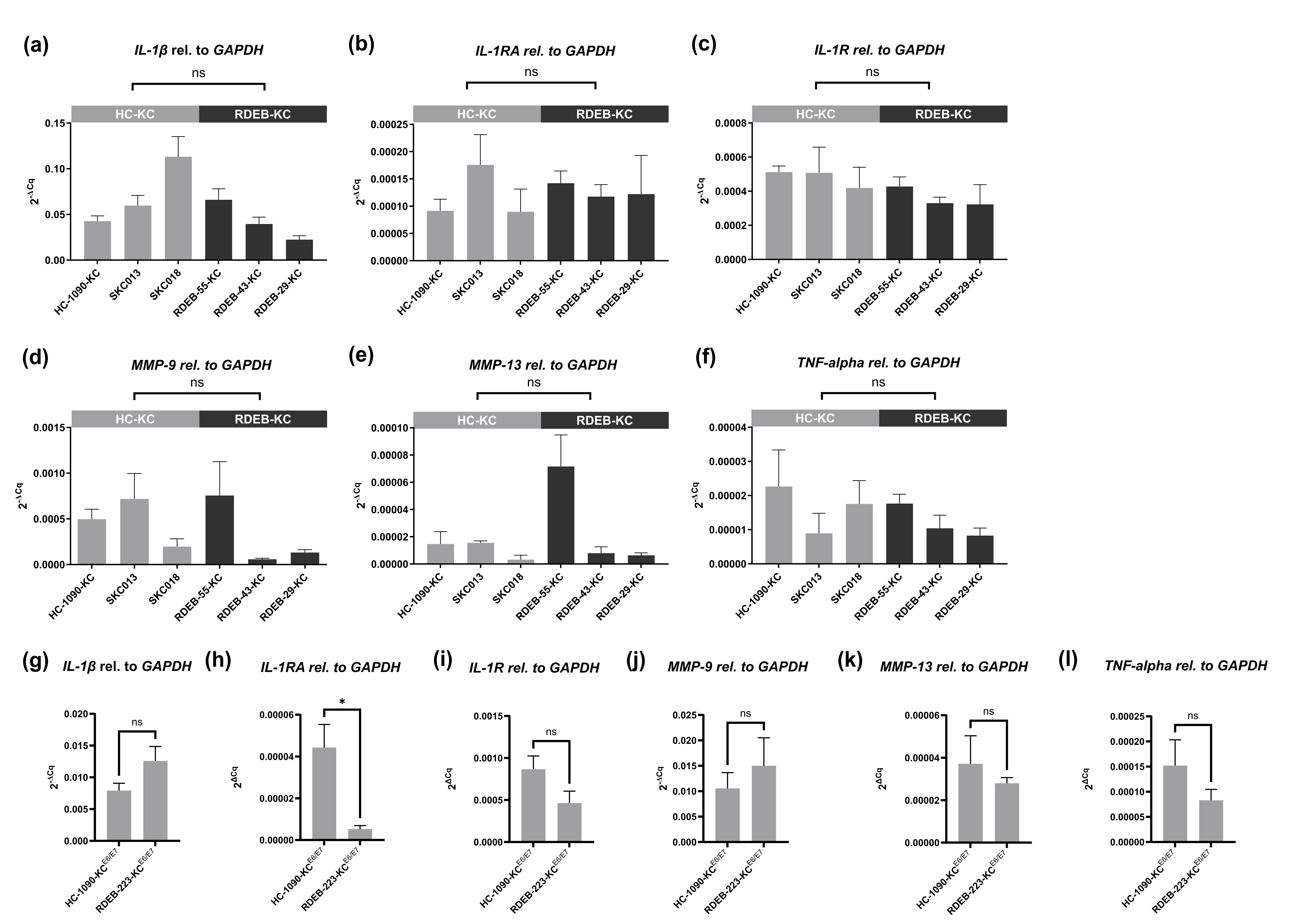


## Fig. S2. IL-1β and MMP-9 expression in RDEB-223-KC^E6/E7^ patient keratinocytes is stimulated by LPS treatment. The expression of IL-1ß and MMP-9 relative to *GAPDH* in RDEB-223-KC^E6/E7^ was analysed by qPCR and is shown as fold change, normalised to untreated control. Bar graphs display mean of fold change with error bars indicating the standard error of mean (SEM) from 4-5 independent biological replicates. Statistical analysis was performed using the Kruskal-Wallis test, and a p-value of < 0.05 was considered as statistically significant with ns: p > 0.05, *: p ≤ 0.05, **: p ≤ 0.01.


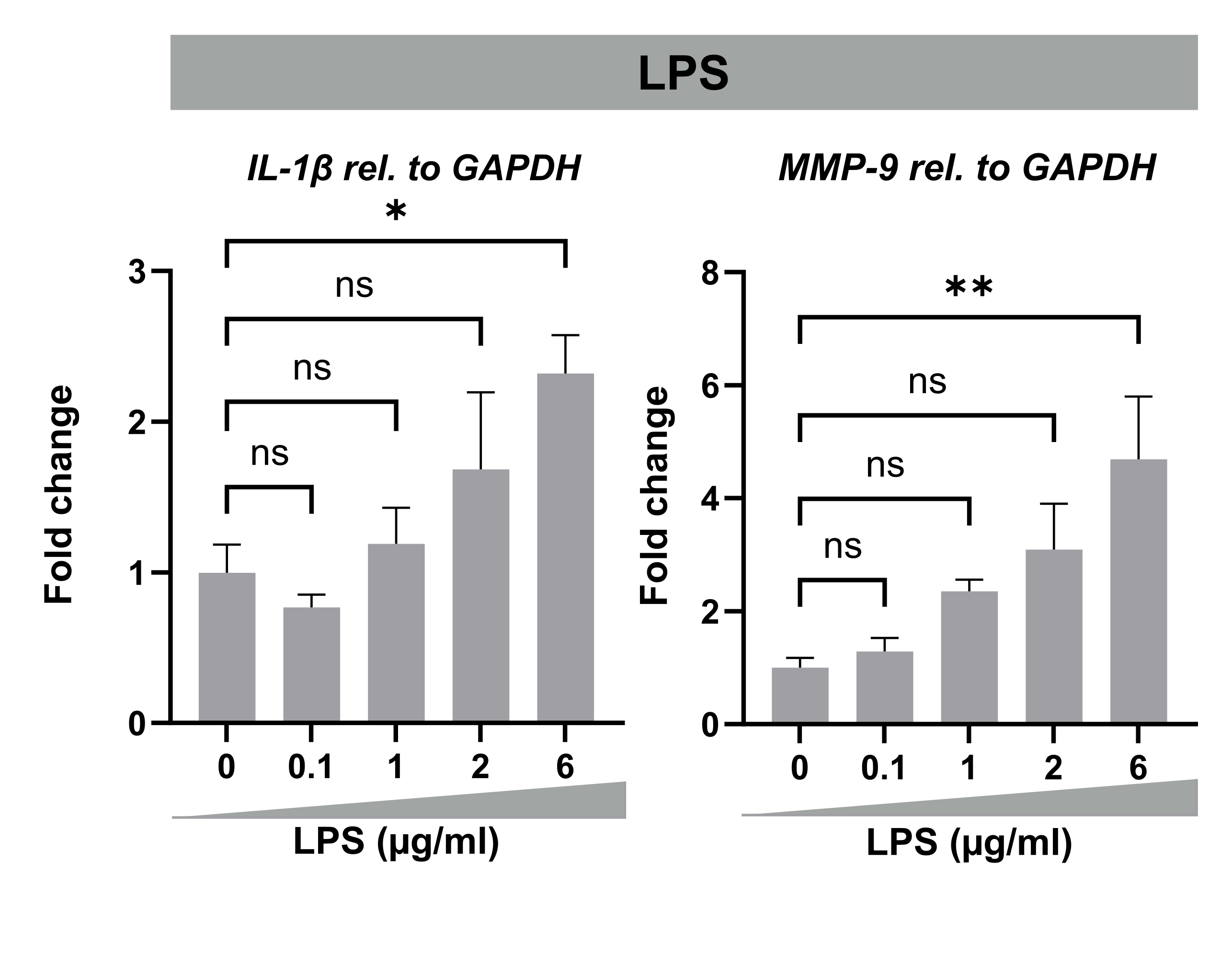


## Fig. S3. IL-1β and MMP-9 expression in RDEB-223-KC^E6/E7^ patient keratinocytes is stimulated by rIL-1ß treatment. The expression of IL-1ß and MMP-9 relative to GAPDH in RDEB-223-KC^E6/E7^ was analysed by qPCR and is shown as fold change, normalised to mock control (0h) for 4 and 24 hours of exposure to 15 ng / mL recombinant IL-1ß (rIL-1ß). Bar graphs display mean of fold change with error bars indicating the standard error of mean (SEM) from 4-5 independent biological replicates. Statistical analysis was performed using the Kruskal-Wallis test. ns: p > 0.05, *: p ≤ 0.05, **: p ≤ 0.01.


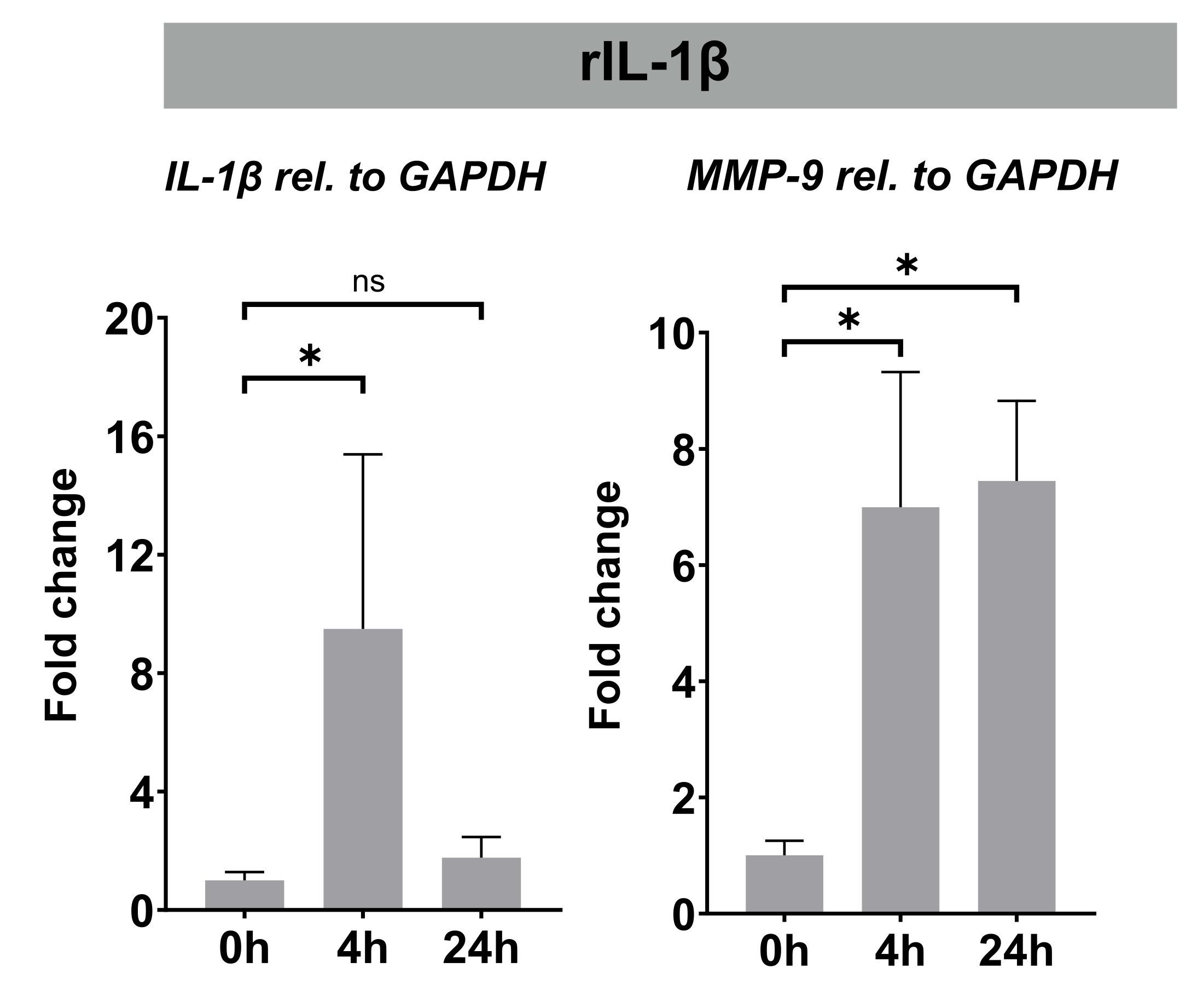


## Fig. S4. Expression of type-VII collagen upon diacerein treatment. Treatment of patient keratinocytes (+) with 10 µg/ml diacerein for 24 hours did not alter overall expression levels of C7 (290kD), compared to untreated RDEB-keratinocytes (-). ß-Tubulin was used as loading control.


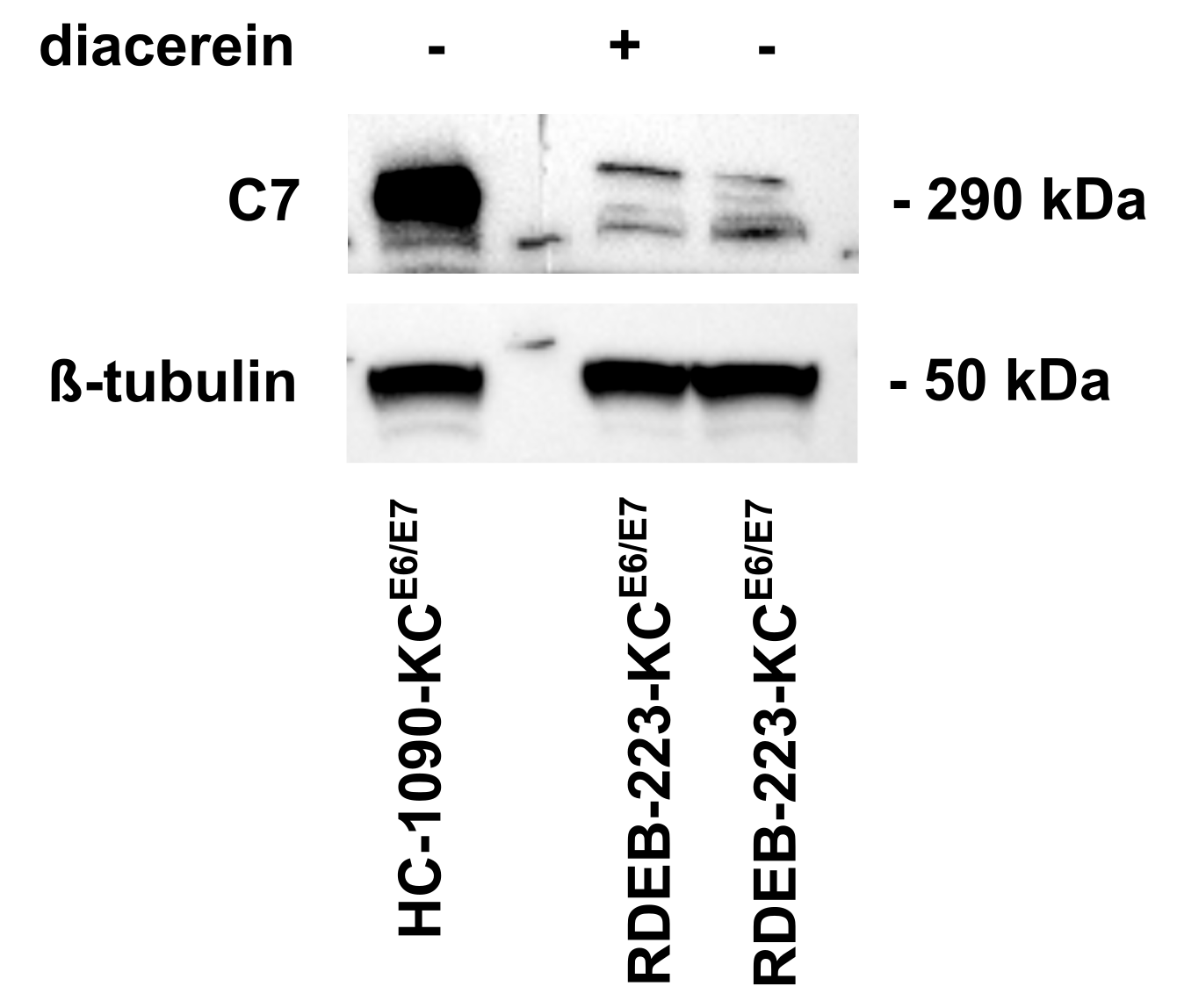


## Fig. S5. Expression profile of IL-1β and MMP-9 in RDEB skin in relation to the wound edge and chronic wound. (a) Immunofluorescence staining for IL-1ß (green) and MMP-9 of denuded skin of a chronic RDEB wound. (b) Relative fluorescence intensity of chronic wound compared to dermis of non-lesional skin and dermal compartment at the wound margin.


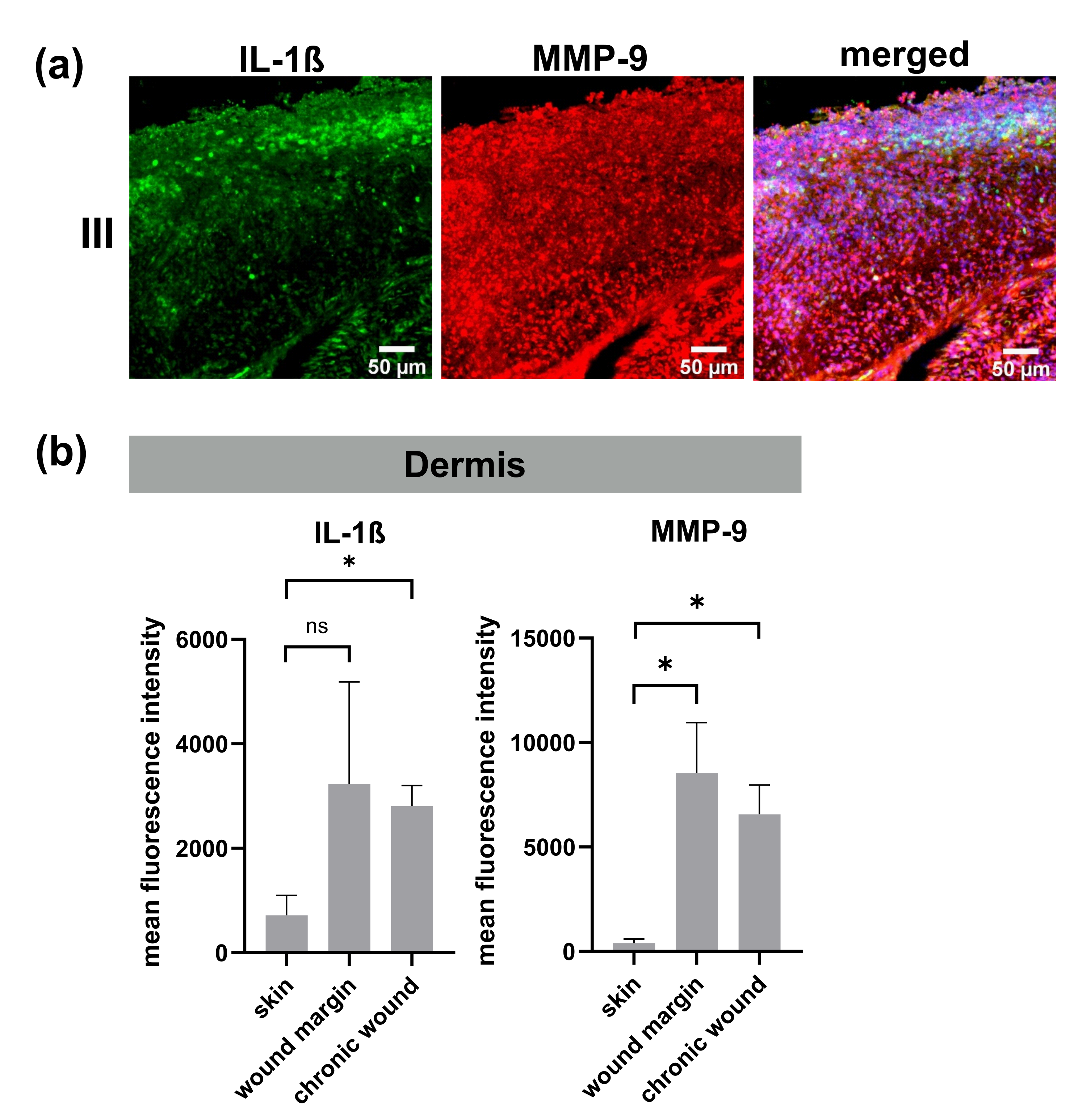


# SUPPLEMENTAL TABLE LEGENDS

## Table SI. Cell lines

| **Cell line** | **Sex** | **Age (years)** | ***COL7A1* mutation (allele 1/allele 2)** | **Localization** |
| --- | --- | --- | --- | --- |
| RDEB-223-KC^E6/E7^ | f | 1 | c.425A>G / c.425A>G | upper thigh |
| HC-1090-KC^E6/E7^ | m | 23 | none | arm |
| SKC013 | f | 35 | none | abdominal |
| SKC018 | m | 31 | none | abdominal |
| HC-1090-KC | m | 23 | none | arm |
| RDEB-55-KC | m | 21 | c.976+4A>C  p.INS34AA | left upper thigh |
| RDEB-43-KC | f | 17 | c.4027C>T / c.425A>G  p.R1343X / p.K142R | right inner thigh |
| RDEB-29-KC | m | 2 | c.425A>G / c.520G>A  p.L142R / p.G174R | foreskin |

## Table SII. Antibodies used for immunofluorescence staining

| **Antibody** | **Host** | **Clone** | **Company** | **Order no.** | **Dilution** |
| --- | --- | --- | --- | --- | --- |
| IL-1β | rabbit | polyclonal | abcam | ab2105 | 1:140 |
| MMP-9 | mouse | 56-2A4 | abcam | ab58803 | 1:280 |
| A488 goat anti rabbit |  |  | Invitrogen |  | 1:400 |
| A594 goat anti mouse |  |  | Invitrogen |  | 1:400 |

## Table SIII. Antibodies used for Western Blot

| **Antibody** | **Host** | **Clone** | **Company** | **Order no.** | **Dilution** |
| --- | --- | --- | --- | --- | --- |
| MMP-9 | rabbit | EP1254 | abcam | ab76003 | 1:1000 |
| hFAB™ Rhodamine Anti-Tubulin Primary Antibody |  |  | Biorad | 12004166 | 1:5000 |
| Goat ANTI-RABBIT IgG StarBright™ Blue 700 |  |  | Biorad | 12004162 | 1:3000 |

## Table SIV. Primer sequences

| **Target** | **Forward primer** | **Reverse primer** |
| --- | --- | --- |
| GAPDH | GCCAACGTGTCAGTGGTGGA | CACCACCCTGTTGCTGTAGC |
| MMP-9 | GGTGTCGCGGAGCACGG | GAGTTGGAACCACGACGCC |
| IL-1ß | GTCCTGCGTGTTGAAAGATGATAAGCC | TTGAGAGGTGCTGATGTACCAGTTGG |
| MMP-13 | TTGAGCTGGACTCATTGTCG | GGAGCCTCTCAGTCATGGAG |
| IL-1RA | CCTCAGAAGACCTCCTGTCCT | GTCGGCAGATCGTCTCCTT |
| IL-1R | ACTGATTTCTTCTCTGGAGGCTG | GGGTTAAGAGGACAGGGACG |
| TNF-alpha | CCCATGTTGTAGCAAACCCT | TGAGGTACAGGCCCTCTGAT |

GAPDH: Glyceraldehyde 3-phosphate dehydrogenase; MMP-9: Matrix metallopeptidase 9; IL-1ß: Interleukin-1β; MMP-13: Matrix metallopeptidase 13; IL-1RA: interleukin-1 receptor antagonist; IL-1R: Interleukin 1 receptor; TNF-Alpha: Tumour Necrosis Factor alpha
